# Idiopathic Bilateral Internal Jugular Vein Thrombosis Diagnosed by Point-of-Care Ultrasound

**DOI:** 10.5811/cpcem.2019.11.44855

**Published:** 2020-01-21

**Authors:** Victor M. Aquino-Jose, Jennifer Johnson, Tina Dulani

**Affiliations:** * Stony Brook University Hospital, Department of Surgery, Division of Trauma, Emergency Surgery, and Critical Care, Stony Brook, New York; † North Shore University Hospital-Northwell Health, Department of Emergency Medicine, Manhasset, New York; ‡ Donald and Barbara Zucker School of Medicine at Hofstra/Northwell, Department of Emergency Medicine, Manhasset, New York

## Abstract

Internal jugular vein (IJV) thrombosis is an unusual condition, especially when it develops bilaterally. This is a case of bilateral IJV thrombosis in a 77-year old female who presented to the emergency department with neck and arm swelling after discontinuing apixaban and undergoing an oropharyngeal procedure. The diagnosis of bilateral IJV thrombosis was made with the use of point-of-care ultrasound to evaluate bilateral jugular vein distention and bilateral upper extremity pitting edema found on her physical examination.

## CASE PRESENTATION

A 77-year-old female with a history of atrial fibrillation currently taking apixaban presented to the emergency department (ED) with swelling of her neck and arms for the past seven days. Two weeks prior, she underwent an embolization of a greater palatine pseudoaneurysm, for which she stopped taking apixaban. After being discharged from the hospital, she continued to experience neck swelling, and had an upper extremity ultrasound evaluation which was negative for thrombus. She returned to the ED for worsening neck swelling. She denied shortness of breath, chest pain, fever, or a history of thromboembolic disease. Her physical exam showed pitting edema on bilateral upper extremities and bilateral jugular vein distention. Point-of-care ultrasound was performed on patient’s neck and upper extremities, revealing thrombosis in bilateral internal jugular veins (IJV) as seen in the Image. The patient was started on anticoagulation and admitted to the Intensive Care Unit (ICU) for monitoring due to significant thrombus burden.

## DISCUSSION

IJV thrombosis is an uncommon condition and very rare when it occurs bilaterally. Previous studies indicate that it usually occurs in patients with a history of malignancy, oropharyngeal infections, or deep vein thrombosis.^1^,^2^ In this case, the patient’s recent oropharyngeal procedure and cessation of apixaban may have led to thrombus formation. IJV thrombosis increases the risk of clot migration and further thrombosis, leading to pulmonary embolism and cerebral vein thrombosis.^3^ These risks increase when there is bilateral IJV thrombosis due to significant venous outflow obstruction.^4^ Patients with this condition should be started on anticoagulation and monitored for signs of clot migration or worsening thrombosis. The patient was admitted to the ICU for initial monitoring while on anticoagulation due to significant clot burden and was later discharged home on oral anticoagulation without complications.

**CPC-EM Capsule**

What do we already know about this clinical entity?

Internal jugular vein (IJV) thrombosis may develop in patients with malignancy or oropharyngeal infections.

What is the major impact of the image(s)?

IJV thrombosis may develop bilaterally in patients presenting with jugular vein distention without exhibiting the common causes of bilateral thrombosis of the internal jugular veins.

How might this improve emergency medicine practice?

Point-of-care ultrasound is effective at providing a prompt diagnosis of bilateral IJV thrombosis and should be monitored after anticoagulation is provided.

## Supplementary Information

Video. A) Thrombus in the right internal jugular vein (arrow) seen on transverse view. B) Sagittal view of right internal jugular vein thrombus (arrow) with color doppler showing obstruction of venous outflow and turbulence proximal to the thrombus. C) Thrombus in the distal left internal jugular vein (arrow) seen on transverse view. D) Color doppler of the left internal jugular vein thrombus (arrow) showing partial occlusion of the lumen seen on transverse view.

## Figures and Tables

**Image:**
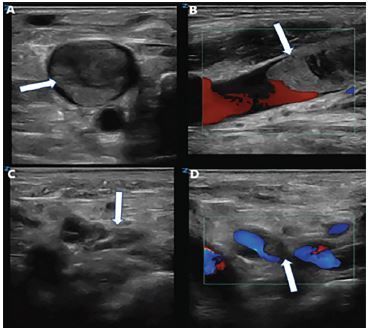
A) Thrombus in the right internal jugular vein (arrow) seen on transverse view. B) Sagittal view of right internal jugular vein thrombus (arrow) with color doppler showing obstruction of venous outflow and turbulence proximal to the thrombus. C) Thrombus in the distal left internal jugular vein (arrow) seen on transverse view. D) Color doppler of the left internal jugular vein thrombus (arrow) showing partial occlusion of the lumen seen on transverse view.
